# A Case of a Giant Siliconoma Mimicking Localized Breast Cancer

**DOI:** 10.5334/jbsr.3691

**Published:** 2024-08-23

**Authors:** Virginie Van Boeckel, Denis Danthine, Jean-Luc Nizet

**Affiliations:** 1 Department of Plastic, Reconstructive and Maxillofacial Surgery, University Hospital of Liège (CHU Liège), Liège, Belgium; 2 Department of Diagnostic and Interventional Radiology, University Hospital of Liège (CHU Liège), Liège, Belgium; 3 Department of Plastic, Reconstructive and Maxillofacial Surgery, University Hospital of Liège (CHU Liège), Liège, Belgium

**Keywords:** Siliconoma, breast, prosthesis, breast augmentation

## Abstract

Silicone gel–filled breast implants are widely used for breast augmentation and reconstruction after mastectomy. However, there are some known complications associated with silicone implants: Leakage and migration of silicone particles from the implant cause a granulomatous reaction. Granulomas may present as masses with features of malignancy on breast MRI. We present a case of a giant breast siliconoma in a woman who had undergone reconstruction with breast prostheses, which were surgically removed because of rupture 8 years ago.

*Teaching point:* Despite increasingly efficient diagnostic tools, siliconoma diagnosis remains challenging.

## Introduction

Silicone breast implants are frequently used for breast augmentation and reconstruction. Localized and systemic complications of silicone implants have been reported, and silicone “bleeding” has been described as occurring from both ruptured and intact implants [1]. This leaking can induce granulomas or, as more recently reported, generalized and poorly differentiated autoimmune inflammatory reactions, also referred to as autoimmune/inflammatory syndrome induced by adjuvants (ASIA) [2]. We present a challenging case of a woman with a past medical history of removed breast prostheses who developed a right breast siliconoma that was surgically removed due to imaging findings mimicking malignancy.

## Case Report

**Figure 1:**
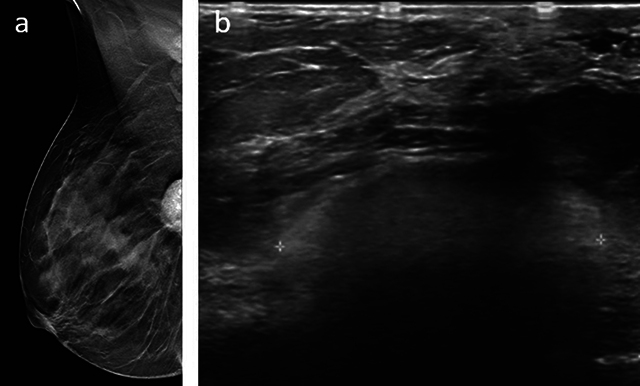
**a.** Right mammogram (mediolateral oblique view): partially visible oval, well-circumscribed deep mass. **b.** Ultrasonography of the right breast: deep, well-circumscribed hyperechogenic mass with typical “snowstorm.”

**Figure 2:**
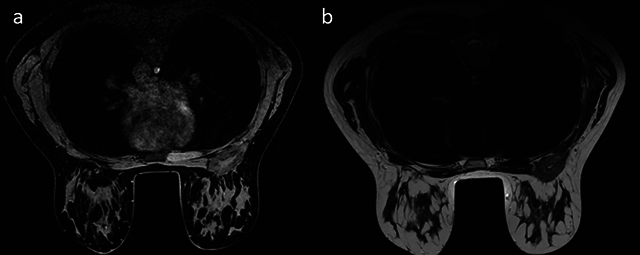
**a.** MRI T1-weighted Dixon water image. **b.** T2-weighted image. Deep right upper mammary lesion, at least partially intra-pectoral, oblong in shape, with irregular margin.

A 43-year-old woman underwent retropectoral breast augmentation 25 years ago, followed by both implants’ removal and a bilateral mastopexy due to the right implant’s rupture 8 years ago. The patient didn’t participate in a breast screening mammogram for several years. A clinical exam with a recent mammography, with a mediolateral oblique view of the right breast, showed an oval, well-circumscribed mass measuring 3 cm in diameter in the upper outer quadrant, which was partially visible due to its location (Figure 1). Ultrasound of the right breast showed a deep oval hyperechoic mass with a “snowstorm” appearance (Figure 1). Magnetic resonance imaging (MRI) showed a corresponding non-enhancing oval mass with irregular margin, partially located in the major pectoralis muscle. The periphery of the lesion had a T2 isosignal and a T1 dixon water iso/slightly hyposignal, whereas the central region showed a T2 hyposignal and a T1 dixon water moderate hypersignal (Figure 2).

Given the medical history, this was presumed to be a siliconoma, albeit one with an atypical MRI presentation. Based on a second reading of the MRI images in our hospital, breast cancer and a fibrous pseudotumour were suggested. Preoperative ultrasound-guided core biopsy revealed multinucleated giant cells with clear vacuoles, consistent with a silicone-associated giant cell, foreign-body reaction.

**Figure 3:**
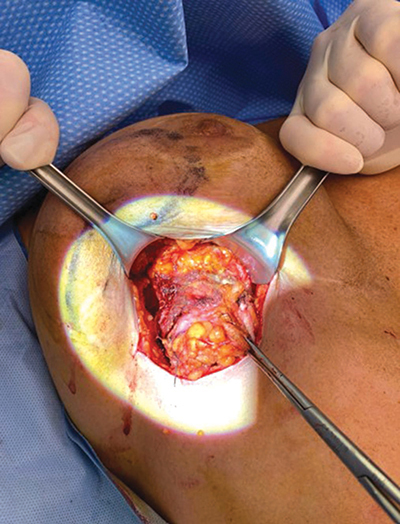
Intraoperative finding after exploration.

**Figure 4:**
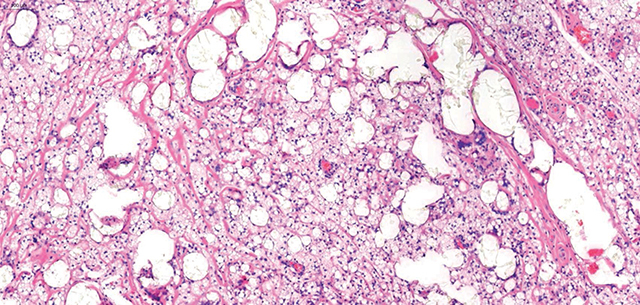
HEx100: Typical multiple silicone vacuoles with macrophages and numerous multinucleate giant cells within the breast lobules and stroma.

The patient underwent tumorectomy after preoperative harpoon tracking. A lump lesion of 51 g was removed, with superficial resection of the pectoral muscle (Figure 3). Pathology was highly suggestive of siliconoma (Figure 4). The post-operative course was uncomplicated.

## Discussion

Even with recent improvements, implant rupture with silicone migration rates remains significant [3]. Once the silicone passes through the shell of the prosthesis, either when ruptured or by continuous perspiration, a granulomatous inflammatory reaction is provoked [4]. However, the migration of silicone through the lymphatic vessels is slow and can take up to 6 to –10 years to reach the lymph nodes. This explains the latency period between the implantation of prostheses and the onset of symptoms or incidental image findings [5].

Although the “snowstorm appearance” is the most accurate and specific sign in ultrasound imaging of extravagated silicone, it can also appear as a complex cystic lesion [6]. This variant likely depends on the amount of extravagated silicone gel, the degree of fibrous and foreign-body reaction, and the elapsed time.

An excisional biopsy is often required in combination with other modalities for definitive diagnosis. Characteristic histologic findings include foamy macrophages and refractile droplets of clear material [7]. Asymptomatic breast siliconoma can be monitored; however, when there is the presence of symptomatic siliconomas with skin involvement, intervention should be undertaken when the patient is eligible for it [5]. Surgical removal of siliconomas also prevents their concealment during future cancer screening and workup.

## Conclusion

The management of a siliconoma is difficult from both the medical and the surgical perspectives. Pathological tissue specimens are the gold standard for diagnosis of siliconomas. There are no established guidelines, although excluding malignancy is essential. In this case, the history of reconstruction by breast prosthesis and removal due to leakage was key to the diagnosis.
